# Nociplastic Pain Criteria or Recognition of Central Sensitization? Pain Phenotyping in the Past, Present and Future

**DOI:** 10.3390/jcm10153203

**Published:** 2021-07-21

**Authors:** Jo Nijs, Astrid Lahousse, Eleni Kapreli, Paraskevi Bilika, İsmail Saraçoğlu, Anneleen Malfliet, Iris Coppieters, Liesbet De Baets, Laurence Leysen, Eva Roose, Jacqui Clark, Lennard Voogt, Eva Huysmans

**Affiliations:** 1 Pain in Motion Research Group (PAIN), Department of Physiotherapy, Human Physiology and Anatomy, Faculty of Physical Education & Physiotherapy, Vrije Universiteit Brussel, 1050 Brussels, Belgium; Astrid.Lucie.Lahousse@vub.be (A.L.); anneleen.malfliet@vub.be (A.M.); iris.coppieters@vub.be (I.C.); liesbet.de.baets@vub.be (L.D.B.); Laurence.Leysen@vub.be (L.L.); Eva.Charlotte.S.Roose@vub.be (E.R.); Jacqueline.Clark@vub.be (J.C.); l.p.voogt@hr.nl (L.V.); eva.huysmans@vub.be (E.H.); 2 Chronic Pain Rehabilitation, Department of Physical Medicine and Physiotherapy, University Hospital Brussels, 1050 Brussels, Belgium; 3 Unit of Physiotherapy, Department of Health and Rehabilitation, Institute of Neuroscience and Physiology, University of Gothenburg Center for Person-Centred Care (GPCC), Sahlgrenska Academy, University of Gothenburg, 405 30 Gothenburg, Sweden; 4 Research Foundation—Flanders (FWO), 1000 Brussels, Belgium; 5 Clinical Exercise Physiology & Rehabilitation Research Laboratory, Physiotherapy Department, Faculty of Health Sciences, University of Thessaly, 382 21 Lamia, Greece; ekapreli@uth.gr (E.K.); pbilika@uth.gr (P.B.); 6 Kütahya Health Sciences University, Kütahya 43100, Turkey; ismail.saracoglu@ksbu.edu.tr; 7 Pains and Brains, Specialist Pain Physiotherapy Clinic, New Plymouth 4310, New Zealand; 8 University of Applied Sciences Rotterdam, 3015 Rotterdam, The Netherlands

**Keywords:** nociplastic pain, precision medicine, neuropathic, central sensitization, nociceptive

## Abstract

Recently, the International Association for the Study of Pain (IASP) released clinical criteria and a grading system for nociplastic pain affecting the musculoskeletal system. These criteria replaced the 2014 clinical criteria for predominant central sensitization (CS) pain and accounted for clinicians’ need to identify (early) and correctly classify patients having chronic pain according to the pain phenotype. Still, clinicians and researchers can become confused by the multitude of terms and the variety of clinical criteria available. Therefore, this paper aims at (1) providing an overview of what preceded the IASP criteria for nociplastic pain (‘the past’); (2) explaining the new IASP criteria for nociplastic pain in comparison with the 2014 clinical criteria for predominant CS pain (‘the present’); and (3) highlighting key areas for future implementation and research work in this area (‘the future’). It is explained that the 2021 IASP clinical criteria for nociplastic pain are in line with the 2014 clinical criteria for predominant CS pain but are more robust, comprehensive, better developed and hold more potential. Therefore, the 2021 IASP clinical criteria for nociplastic pain are important steps towards precision pain medicine, yet studies examining the clinimetric and psychometric properties of the criteria are urgently needed.

## 1. Introduction

Chronic pain is the most prevalent disease worldwide, leading to substantial disability and enormous socioeconomic burden [1]. Amongst long-term conditions, it is responsible for the highest number of years lived with disability [2,3] and is the most expensive cause of work-related disability [4,5]. Thus, chronic pain can be regarded as a non-communicable disease with a large impact on public health. Chronic pain is often non-specific, implying that there is no pathology or tissue damage or that the limited amount of pathology or tissue damage is not severe enough to explain the pain experience. This non-specific nature accounts for non-cancer pain as well as post-cancer pain (i.e., pain in cancer survivors) [6].

In many people with chronic non-specific pain, central nervous system sensitization (briefly: central sensitization or CS; the Appendix A provides a terminology overview) can explain why they suffer from pain in the absence of a clear origin of nociceptive input or in the absence of enough tissue damage to explain the experienced pain severity, disability and other symptoms [7,8]. For clinical purposes, CS is defined as an amplification of neural signalling within the central nervous system that elicits pain hypersensitivity [7]. Under this definition, it is possible to study CS in humans. However, this is not the case for the definition provided by the International Association for the Study of Pain (IASP): “an increased responsiveness of nociceptive neurons in the central nervous system to their normal or subthreshold afferent input” [9] because in vivo measurements of nociceptive neuron responses in the central nervous system are impossible. CS encompasses various related dysfunctions within the central nervous system, including altered sensory processing in the brain [10] with a disrupted resting state functional connectivity in the default mode and salience networks [11] and increased brain activity in areas known to be involved in acute pain sensations (insula, anterior cingulate cortex and prefrontal cortex) as well as in other regions (various brain stem nuclei, dorsolateral frontal cortex and parietal associated cortex) [12]. CS also includes altered activity in brain-orchestrated nociceptive facilitatory pathways [10,13]. CS also implies poor functioning of endogenous analgesia (Appendix A), which refers to brainstem-originated pathways that release neurotransmitters to inhibit spinal nociceptive processing [14,15]. Together, these central nervous system dysfunctions not only contribute to increased responsiveness to a variety of sensory inputs such as tactile stimuli but can also lead to hypersensitivity to non-musculoskeletal stimuli, such as chemical substances, light, sound, heat, cold, stress and electricity [16]. The knowledge regarding CS has revealed a paradigm shift in the understanding and management of chronic pain that allows clinicians to think beyond muscles and joints and to account for the role of pain modulation in the central nervous system [17].

In a variety of chronic musculoskeletal pain conditions, CS has been found to be present in an important subgroup of patients (reviewed in [17]). These conditions include chronic traumatic neck pain (i.e., whiplash) [18], fibromyalgia [19], osteoarthritis [20], migraine [21], irritable bowel syndrome [22], chronic fatigue syndrome [23], paediatric pain [24], low back pain [25], non-traumatic neck pain [26], rheumatoid arthritis [27] and pain following cancer [6]. CS appears to be less common in patients with tennis elbow [28], tendinopathies [29] and shoulder pain [30]. This illustrates the need for clinical recognition of CS in individual patients with chronic pain. Indeed, in conditions, such as tendinopathies, where CS is present in a minority of patients, the clinical importance of CS is illustrated by studies showing that the subgroup of the population characterized by CS is more disabled and suffers more severe pain than those who do not have CS [28,31]. Additionally, the presence of (symptoms of) CS predicts poor treatment outcomes in patients with a variety of chronic pain conditions [32,33,34,35,36], at least when the treatment targets the presumed source of nociception. This applies to conservative interventions [35,36] but also to surgical interventions [37,38,39,40]. Again, this shows the need for early recognition of CS in patients with chronic pain, in combination with tailored treatment [41].

This need for early recognition of CS in patients with chronic pain was picked up by IASP, who introduced the term “nociplastic pain” in 2017 as a third mechanistic pain descriptor in addition to nociceptive and neuropathic pain (Appendix A) [42,43]. Nociplastic pain is defined by the IASP as “pain that arises from altered nociception despite no clear evidence of actual or threatened tissue damage causing the activation of peripheral nociceptors or evidence for disease or lesion of the somatosensory system causing the pain” (Appendix A) [43]. CS is not part of the definition of nociplastic pain; however, signs of sensitization are generally present in nociplastic pain conditions [42]. Moreover, sensitization is the major underlying mechanism of nociplastic pain [44]. Hence, patients whose clinical picture is dominated by CS are labelled as having nociplastic pain. Recently, the IASP released clinical criteria and a grading system for nociplastic pain affecting the musculoskeletal system [44]. These criteria replaced the 2014 clinical criteria for predominant CS pain [45] and are embraced by the international community, as they account for clinicians’ need to (early) identify and correctly classify patients having chronic pain according to the pain phenotype. Still, clinicians and researchers can become confused by the multitude of terms (CS, predominant CS pain, nociplastic pain, central sensitivity syndromes, etc.) and the variety of clinical criteria available. Therefore, the present paper aims at (1) providing an overview of what preceded the IASP criteria for nociplastic pain (‘the past’); (2) explaining the new IASP criteria for nociplastic pain in comparison with the 2014 clinical criteria for predominant CS pain (‘the present’); and (3) highlighting key areas for future implementation and research work in this area (‘the future’).

## 2. The Past

The first scientific reports that addressed the issue of clinical identification of CS pain in patients with chronic musculoskeletal pain date back to 2010 [16,46]. A ‘masterclass’ paper described an initial attempt for supporting clinicians in their clinical reasoning process to recognize CS in patients with chronic musculoskeletal pain [16], but the first structured approach to adding CS pain to pain phenotyping in clinical practice was provided in 2010 by Keith Smart et al. [46]. They reported an expert consensus-derived list of clinical criteria suggestive of a clinical dominance of nociceptive, peripheral neuropathic and ‘central’ mechanisms of musculoskeletal pain [46]. This pioneering work was expanded with the same group reporting a study of 64 patients with low back and leg pain, where they identified key symptoms that allowed clinicians to differentiate with a high discriminative ability [47] CS pain from nociceptive and neuropathic pain [48]: disproportionate, non-mechanical, unpredictable pattern of pain provocation in response to multiple/non-specific aggravating/easing factors, pain disproportionate to the nature and extent of injury or pathology, strong association with maladaptive psychosocial factors and diffuse/non-anatomic areas of pain/tenderness on palpation. 

Based on the work by Smart et al. [46,47], the available literature at that time and expert consensus, clinical criteria for recognizing predominant CS in patients with chronic musculoskeletal pain were released in 2014 [45]. These recommendations included the exclusion of neuropathic pain as the first step. A second mandatory criterion involved determining if the severe pain can be considered disproportionate to what one would expect based on the available tissue damage or presumed source of nociception [45]. When neuropathic pain is not present and the pain is considered disproportionate in nature, at least one of the two remaining criteria should be met. The first of the two optional criteria is the diffuse pain distribution, i.e., pain that spreads outside the segmental area of primary nociception [45,49]. Applied to the example of knee osteoarthritis, this criterion corresponds to someone having pain referring all over the affected low limb [49]. Pain drawings can be used to standardize and optimize the assessment of the individual’s pain distribution in a reliable way. Patients are requested to shade the areas where they experience pain on an outline of a human figure. The human figure is divided into 45 body areas. Each body part is equal to a certain percentage of the total body surface [50]. The more areas are shaded, the more indication for widespread pain. The data obtained using this tool show acceptable test-retest reliability (r = 0.85) [51]. Furthermore, two-dimensional computerized methods have been developed for more precise measurement of pain extent, making interpretation more standardized, presenting excellent intra-rater and inter-rater reliability (ICC = 0.99–0.97) [52]. The second optional criterion for predominant CS encompasses a score of 40 or higher on the Central Sensitization Inventory (CSI). The CSI is a patient-reported outcome measure that has been widely used to study symptoms of CS in patients with chronic pain. It assesses pain as well as non-pain symptoms considered to be related to CS (e.g., unrefreshing sleep, sleep problems, sensitivity to light, concentration difficulties, stress as aggravating factor, sensitivity to odours, restless legs) [53]. The CSI is available in 18 languages and can be accessed free of charge at » Questionnaires Developed at PRIDE (pridedallas.com, accessed on 9 June 2021). The psychometric properties of the CSI in patients having non-specific, non-cancer pain are well-established [54]. Taken together, the 2014 clinical criteria for predominant CS pain suggested that one can expect the presence of CS in case of a disproportional pain experience combined with a diffuse pain picture and/or a score above 40/100 on the CSI [45]. Furthermore, the relationship of the CSI to biopsychosocial aspects of CS pain may be supported based on findings that the extent of CS symptoms, measured using the CSI in chronic low back pain with clinically identified CS (2014 criteria), can be predicted by trait anxiety and trait sensory hyper-sensitivity characteristics [55].

Following its publication in 2014, the clinical criteria for recognizing predominant CS in patients with chronic musculoskeletal pain [45] were adapted for specific chronic pain populations such as osteoarthritis [56], low back pain [57], chronic pelvic and perineal pain criteria [58] and post-cancer pain [6]. Next, the IASP introduced the term “nociplastic pain” as a third mechanistic pain descriptor [42,43], which brings us to the present situation.

## 3. The Present

The IASP clinical criteria and grading system for nociplastic pain of the musculoskeletal system.

The IASP clinical criteria for nociplastic pain of the musculoskeletal system imply that, in order to clinically classify nociplastic pain, patients have to:

(1) report pain of at least 3 months duration;
(2) report a regional rather than discrete pain distribution;
(3) report pain that cannot entirely be explained by nociceptive or neuropathic mechanisms;
(4) show clinical signs of pain hypersensitivity (i.e., evoked pain hypersensitivity phenomena such as static or dynamic mechanical allodynia, heat or cold allodynia, and/or painful after-sensations after any of the mentioned evoked pain hypersensitivity assessments) that are at least present in the region of pain [44].

**Figure 1:**
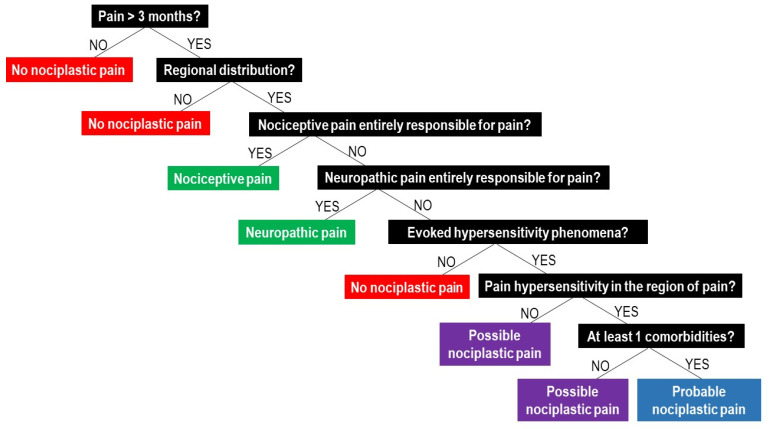
Clinical decision-making tree of the IASP clinical criteria for nociplastic pain.

If these four requirements are met, the patients can be classified as having “possible nociplastic pain” [44]. In cases where all four requirements are fulfilled, plus the patient presents with a history of pain hypersensitivity in the region of pain (i.e., sensitivity to touch, movement, pressure or heat/cold) and at least one of the defined comorbidities (increased sensitivity to sound, light and/or odours, sleep disturbance with frequent nocturnal awakenings, fatigue or cognitive problems), the pain is classified as “probable nociplastic pain” [44]. The presence of nociceptive or neuropathic pain does not exclude the possibility of concurrence of nociplastic pain, but if nociceptive or neuropathic pain is present, they cannot be entirely responsible for the pain. For clinicians willing to apply the IASP clinical criteria for nociplastic pain during their clinical reasoning process, Figure 1 provides a clinical decision-making tree.

The IASP clinical criteria and grading system for nociplastic pain affecting the musculoskeletal system [44] provide the first set of clinical criteria (1) endorsed by a worldwide scientific organization (i.e., the IASP), and (2) linked to nociplastic pain as the third mechanistic pain descriptor in addition to nociceptive and neuropathic pain. CS is a key underlying mechanism of nociplastic pain [44], yet CS goes beyond the nociceptive system [17]. Within this view, it is considered unfortunate that the IASP chose the term nociplastic pain and defined it with a focus on the nociceptive system. The IASP nociplastic pain criteria, however, do appear to account for this shortcoming, as they stress the importance of assessing comorbidities with non-pain symptoms and sensory (rather than nociceptive) hypersensitivity being part of the IASP 2021 criteria [44]. It is key for clinicians to understand that non-pain symptoms can result from the same underlying mechanism (i.e., CS), and the IASP clinical criteria for nociplastic pain facilitate this.

### Comparing the IASP Clinical Criteria for Nociplastic Pain with the 2014 Clinical Criteria for Predominant Central Sensitization Pain

When comparing the 2021 IASP clinical criteria for nociplastic pain of the musculoskeletal system with the 2014 clinical criteria for predominant CS pain in patients with musculoskeletal pain (Table 1), it becomes clear that both sets of criteria focus on chronic pain, which is pain of at least three months’ duration. Additionally, both sets target musculoskeletal pain, implying that they are not intended for pain phenotyping in patients with visceral pain. Looking at the individual criteria, the IASP 2021 criterion of the patient reporting a regional rather than discrete pain distribution [44] is similar to the 2014 criterion of diffuse pain that spreads outside the segmental area of primary nociception [45,49]. The main difference here is that it is a mandatory criterion in the IASP 2021 criteria, while it was an optional criterion in the 2014 criteria. The requirement of excluding predominant neuropathic pain as the underlying mechanism is another agreement between both sets of criteria. In addition, the IASP criteria state that the pain cannot entirely be explained by nociceptive mechanisms [44], which is a much more straightforward way of what was intended in the 2014 criteria with ‘disproportionate pain’ [45]. ‘Disproportionate pain’ may be viewed as a subjective assessment by the therapist, which the 2021 IASP criteria eliminate. Indeed, disproportionate pain was defined as “the severity of pain is disproportionate to the nature and extent of injury or pathology (i.e., tissue damage or structural impairments),” and it was explained that disproportionate pain contradicts nociceptive pain, where the severity of pain is proportionate to the nature and extent of injury or pathology [45]. Hence, both sets of criteria stress the importance of differentiating from nociceptive pain by excluding the possibility that nociception is the main driver of the experienced pain and consequently used this as a mandatory criterion. Of note is also that three out of four of the symptoms identified by Smart et al. in 2012 [48] as having the ability to differentiate between peripheral neuropathic, nociceptive and CS pain are included (in other wordings) in the 2021 IASP clinical criteria for nociplastic pain. It is worth mentioning that more than one pain phenotype can present. Taken together, the 2021 IASP criteria emphasize better the possibility for mixed types of pain.

A major difference between the 2014 and the 2021 IASP criteria is that the latter demand clinical signs of pain hypersensitivity in at least the region of pain evoked during clinical assessments of mechanical, heat or cold allodynia [44]. Adding this as a mandatory criterion makes a lot of sense considering the body of literature regarding sensory hypersensitivity in a variety of patients with nociplastic pain [59,60,61,62], yet it remains to be determined whether the outcome of such clinical tests of allodynia in the region of pain has a discriminative ability with what is seen in patients with nociceptive and neuropathic pain. In fact, primary hyperalgesia is seen also in inflammatory or nociceptive pain, as well as in neuropathic pain [63]. Additionally, in the case of chronic, persistent neuropathic pain, dysfunctions within the central nervous system related to CS may (partly) explain symptoms in these patients [64]. The discriminative ability of allodynia in areas remote from the painful region [65,66,67,68] may be higher, but this is not addressed in either the 2021 IASP or 2014 clinical criteria. Another key difference between the two sets of criteria is the use of a grading system in the 2021 IASP criteria, which was not included in the 2014 criteria. We feel this is a very positive evolution, as it is in line with the approach of grading the likelihood of having neuropathic pain [69] but also as it reflects modesty and the level of evidence supporting the clinical criteria in individual patients. However, the additional requirements needed for grading the pain as probable nociplastic pain partly overlap with the fourth criterion of the 2014 criteria. The 2021 IASP criteria for probable nociplastic pain require that the patient presents with a history of hypersensitivity to touch, movement, pressure or heat/cold in the region of pain and increased sensitivity to sound, light and/or odours, sleep disturbance with frequent nocturnal awakenings, fatigue or cognitive problems [44]. Quantitative sensory testing can be used for assessing hypersensitivity to pressure, heat and cold, but according to the 2021 IASP clinical criteria, this is not mandatory. While a history of hypersensitivity to touch, movement, pressure or heat/cold in the region of pain were not included in the 2014 clinical criteria for predominant CS pain, all the comorbidities of the 2021 IASP clinical criteria for nociplastic pain can be assessed using the CSI (Table 2). Therefore, even though the CSI was not proposed by the 2021 IASP criteria, we believe that in the patient interview, most of the CSI items can assist in querying the history of pain hypersensitivity and non-painful comorbidities. In addition, the CSI can also provide clinicians information regarding the severity of sensory hypersensitivity, as it gives a numerical value. A possible limitation of the CSI, where the CSI score is used to confirm the presence of a predominant CS pain mechanism in the 2014 criteria, is the risk of false negatives, which the IASP 2021 criteria eliminate. For example, CSI scores may be confounded by individual coping styles with characteristics that tend to under-report themselves in subjective measures, which they consider might cast them in a negative light [55]. Taken together, one should keep in mind that the CSI is intended to assess symptoms of CS and is not meant to be a tool to “diagnose” CS. Therefore, it cannot be used as a standalone questionnaire for the identification of predominant CS/nociplastic pain and should always be combined with the assessment of the remaining clinical criteria for the identification of nociplastic pain.

Taken together, the 2021 IASP clinical criteria for nociplastic pain of the musculoskeletal system are in line with the 2014 clinical criteria for predominant CS pain, but they are more comprehensive, better developed and hold more potential due to its support from a large international organization such as the IASP.

## 4. The Future

### 4.1. Towards Precision Pain Medicine?

CS facilitates embracing the biopsychosocial model for the assessment, clinical reasoning and treatment of patients with chronic pain. In addition, clinical criteria for nociplastic pain allow clinicians to adopt the treatment according to the pain phenotype. Precision medicine refers to the ability to classify patients into subgroups that differ in their susceptibility to, biology, or prognosis of a particular disease, or in their response to a specific treatment, and thus to tailor treatment to the individual patient characteristics [70]. Recent studies suggest that assessment of CS may be used to improve precision pain medicine for rheumatology practices (reviewed in [17]). This implies that patient education, management and treatments are adapted to the pain phenotype. For patient education, this includes explaining the underlying pain mechanism to the patient according to the relevant pain phenotype (e.g., explaining central sensitization to patients having nociplastic pain) [71]. For the management and treatment of pain, it implies that injury- and pathology-targeted approaches (i.e., classical biomedical treatments such as surgery, joint treatment and anti-inflammatory drugs) should be preserved to patients having nociceptive pain, while nociplastic pain requires a broader multimodal approach including patient education, behavioural graded activity, stress management, exercise therapy, sleep management, etc. [8,72]. A pitfall for clinicians and patients applying such an approach is that the focus of the treatment relies too much on improving the underlying pain mechanism (i.e., decreasing CS in patients with nociplastic pain). Rather, the focus should be to regain the ability to perform and enjoy the patient’s self-chosen functional activities and hence to improve quality of life. Another pitfall for clinicians applying a pain phenotyping approach is that one might neglect the individual variability within one pain phenotype. Therefore, precision medicine for patients with chronic pain is more than accounting for CS, and it also implies addressing relevant comorbidities, such as insomnia [73] and obesity [74], and lifestyle factors that sustain CS, such as stress [75], physical inactivity [76] and unhealthy diet [77], depending on the individual patient characteristics.

It should be acknowledged that (symptoms of) CS can be present to a varying degree and may therefore also be present in people whose predominant pain type may not be nociplastic pain (e.g., in certain people undergoing total knee arthroplasty or surgery to decompress a spinal nerve). However, in the latter case, accounting for (factors underlying) CS can still be of relevance due to the known unfavourable influence of CS on therapy and/or surgical outcomes (refer to the introduction) [35,36,37,38,39,40]. In that sense, although differentiating between predominant pain types can be very useful for clinical practice, clinicians should avoid developing a tunnel view by using such classification criteria.

To facilitate thorough clinical assessment, a guideline for the biopsychosocial assessment of individuals with chronic pain, including the determination of the predominant pain type, is available [78]. The latter was based on the 2014 criteria but can easily be replaced by the 2021 IASP clinical criteria. We feel that applying such a comprehensive biopsychosocial assessment results in a clear overview of the clinical picture of a patient, facilitating a holistic biopsychosocial approach and decreasing the aforementioned risk for tunnel vision of the clinician.

### 4.2. Research Agenda

Still, it is important to stress that research is needed to examine the reliability and validity of the different sets of clinical criteria for nociplastic pain/predominant CS pain. To serve this purpose, clinical vignettes may be useful. Clinical vignettes are short scenarios that describe a situation (i.e., real cases) in which the reader has the opportunity to submit his or her comments and opinion. Vignettes have been used in social [79] and medical studies for diagnosis, clinical reasoning and disease management [80,81]. Participants typically answer a series of open-ended or closed-ended questions related to the scenarios included in the vignettes [82]. Guidelines are available to ensure internal validity when developing vignettes [83,84]. One of the advantages of vignettes is that they can present situations that are influenced by factors that may not be easily accessible for research purposes in real situations. With this technique, these clinical situations are available to multiple evaluators simultaneously and identically. This provides the opportunity to examine the intra- and interrater reliability of the IASP clinical criteria for nociplastic pain to compare the outcome of the IASP clinical criteria for nociplastic pain with the 2014 clinical criteria for predominant CS pain and to examine the content validity of both sets of criteria. In addition, developing reliable and valid field-testing procedures would be valuable for supporting clinicians in applying pain phenotyping in daily practice.

As mentioned before, an assessment protocol for allodynia of a remote painful area could be of added value for clinicians to distinguish the different pain phenotypes [14]. Quantitative sensory testing (QST) is a widely used method that measures patients’ verbal or behavioural response to quantifiable sensory stimuli, which encompasses broad parameters, such as detection and pain thresholds, temporal summation (TS), and conditioned pain modulation (CPM) [85]. Patients with chronic conditions show imbalanced pain facilitation and pain inhibition [86], which are usually assessed, respectively, by TS, controlling for increasing evoked pain by fixed repetitive stimuli, and CPM, controlling for the ability to reduce evoked pain by a second stimulus [86]. A dysregulated response was observed in conditions such as fibromyalgia [87], temporal mandibular disorders [88], irritable bowel syndrome [88] and osteoarthritis [89]. The strength of these assessments is their ability to predict the magnitude of post-operative pain and the effect of exercise interventions [90,91,92]. Unfortunately, until now, it is not realistic to incorporate an elaborated QST protocol in the clinic. This was also underscored in the paper presenting the 2021 IASP clinical criteria for nociplastic pain [44]. However, there is growing interest in bedside QST protocols, which do not require specialized equipment [85]. Some studies provided promising findings regarding the validity and reliability of such bedside QST protocols; however, further work is required, and future research should assess the feasibility in clinical practice, as well as the added value of such bedside QST protocols to the clinical criteria for nociplastic pain [86].

Another future consideration is that nociplastic pain includes CS within its phenotype, but the 2021 IASP criteria do not currently accommodate aspects of sensory hypo-sensitivity. Aspects of sensory hypo-sensitivity may accompany some nociplastic pain presentations [93,94,95], and this warrants further investigation.

## 5. Conclusions

Pain phenotyping in patients with chronic musculoskeletal pain remains a hot topic. The initial attempts to develop clinical criteria for patients having a predominant CS type of pain date back to 2010. In 2017, the IASP introduced the term “nociplastic pain” as a third mechanistic pain descriptor in addition to nociceptive and neuropathic pain, providing a label to patients having a predominant CS type of pain. Recently, the IASP released clinical criteria and a grading system for chronic nociplastic pain of the musculoskeletal system. These 2021 IASP clinical criteria for nociplastic pain of the musculoskeletal system are in line with the 2014 clinical criteria for predominant CS pain but are more robust, comprehensive, better developed and hold more potential due to its support from the IASP. Therefore, the 2021 IASP clinical criteria for nociplastic pain of the musculoskeletal system are an important step towards precision pain medicine, yet studies examining the clinimetric and psychometric properties of the criteria are urgently needed.

## Figures and Tables

**Table 1:** A comparison of the IASP 2021 clinical criteria for nociplastic pain with the 2014 clinical criteria for predominant central sensitization pain.

IASP 2021 Clinical Criteria for Nociplastic Pain [44]	2014 Clinical Criteria for Predominant Central Sensitization Pain [45]
*Mandatory criteria*	
Patients have to report pain of at least 3 months duration.	Patients have to report pain of at least 3 months duration.
Patients have to report a regional rather than discrete pain distribution.	Patients have to present diffuse pain that spreads outside the segmental area of primary nociception.
Patients have to report pain that cannot entirely be explained by nociceptive mechanisms.	The pain should be considered disproportionate to what one would expect based on the available tissue damage or presumed source of nociception.
Patients have to report pain that cannot entirely be explained by neuropathic mechanisms.	Exclusion of neuropathic pain as the dominant pain mechanism.
Patients have to show clinical signs of pain hypersensitivity (i.e., evoked pain hypersensitivity phenomena such as static or dynamic mechanical allodynia, heat or cold allodynia, and/or painful after-sensations after any of the mentioned evoked pain hypersensitivity assessments) that are present at least in the region of pain.	-
*Optional criteria*	
Patients present with a history of pain hypersensitivity in the region of pain (i.e., sensitivity to touch, movement, pressure or heat/cold).	-
Patients present at least one of the defined comorbidities (increased sensitivity to sound, light and/or odours, sleep disturbance with frequent nocturnal awakenings, fatigue or cognitive problems).	A score of at least 40/100 on the Central Sensitization Inventory.

**Table 2:** The comorbidities included in the 2021 IASP clinical criteria for nociplastic pain are covered by items included in the Central Sensitization Inventory. Q = question number.

Comorbidities Included in the 2021 IASP Clinical Criteria for Nociplastic Pain	Central Sensitization Inventory Items
Increased sensitivity to light and/or sound and/or odours	I am sensitive to bright lights (Q7). Certain smells, such as perfumes, make me feel dizzy and nauseated (Q20).
Sleep disturbance with frequent nocturnal awakenings	I feel tired and unrefreshed when I wake up from sleeping (Q1). I do not sleep well (Q12). My legs feel uncomfortable and restless when I am trying to go to sleep at night (Q22).
Fatigue	I get tired very easily when I am physically active (Q8). I have low energy (Q17).
Cognitive problems such as difficulty to focus attention, memory disturbances, etc.	I have difficulty concentrating (Q13). I have difficulty remembering things (Q23).

## Data Availability

Not applicable.

## Appendix A. Terminology (Presented in Alphabetic Order)

**Term**	**Explanation**
Central sensitization	A neurophysiological mechanism, defined as ‘amplification of neural signalling within the central nervous system that elicits pain hypersensitivity [7],’ potentially explains chronic, nonspecific pain.
Central nervous system sensitization	Refers to ‘central sensitization.’
Chronic pain	Pain of at least 3 months duration.
Endogenous analgesia	The body’s ability to activate pain relief, with poor endogenous analgesia considered a feature of central sensitization [17].
Neuropathic pain	Pain due to a lesion or a disease of the nervous system.
Nociceptive pain	Pain due to damage to non-neural tissue (e.g., musculoskeletal or visceral tissue).
Nociplastic pain	Pain that arises from altered nociception despite no clear evidence of actual or threatened tissue damage causing the activation of peripheral nociceptors or evidence for disease or lesion of the somatosensory system causing the pain [43].
Nonspecific pain	Pain that cannot be explained by tissue damage, pathology or local dysfunctions.
